# Supplementary material for: Pan-cancer analysis of PSCA that is associated with immune infiltration and affects patient prognosis
Source: PLoS One. 2024 Jun 25;19(6):e0298469. doi: 10.1371/journal.pone.0298469 (PMC11198779; doi:10.1371/journal.pone.0298469)
Supplement: S1 Fig — (A) DNA methylation-based stemness score (DNAss); (B) RNA-based stemness score (RNAss); (C) Differential methylation probe-based stemness score (DMPss); (D) Enhancer element/DNA methylation-based stemness score (ENHss); (E) Epigenetically regulated RNA-based stemness score (EREG.EXPss); (F) Epigenetically regulated DNA methylation-based stemness score (EREG-METHss). (DOCX) [file pone.0298469.s001.docx]

**Supplemental information for**

Pan-cancer analysis of PSCA, which is associated with immune infiltration and affects patient prognosis

Chenxing Wang^2,6,a^, Xingjia Zhu^2,6,a^, Ming Zheng^3,6,a^, Yixun Chen^4,5^, Rui Jiang^2 ,6^, Xinyu He^5^ Zongheng Liu^2,5,6^, Zhichao Lu^2,5^, Ziheng Wang^5,*^, Yang Yang^1,*^

^1^ Department of Trauma Center, Affiliated Hospital of Nantong University, Medical school of Nantong University, Nantong, 226001, China.

^2^ Department of Neurosurgery, Affiliated Hospital of Nantong University, Medical school of Nantong University, Nantong, 226001, China.

^3^ Department of Laboratory Medicine, Affiliated Hospital of Nantong University, Medical school of Nantong University, Nantong, 226001, China.

^4^ Eye Institute, Affiliated Hospital of Nantong University, Medical School of Nantong University, Nantong 226001, Jiangsu, China

^5^ Department of Clinical Biobank & Institute of Oncology, Affiliated Hospital of Nantong University, Medical school of Nantong University, Nantong, 226001, China.

^6^ Research Center of Clinical Medicine, Affiliated Hospital of Nantong University, Medical school of Nantong University, Nantong, 226001, China.

**Fig. S1 Relationship between PSCA and six tumour stemness indices in LUAD. (A)** DNA methylation-based stemness score (DNAss); **(B)** RNA-based stemness score (RNAss); **(C)** Differential methylation probe-based stemness score (DMPss); **(D)** Enhancer element/DNA methylation-based stemness score (ENHss); **(E)** Epigenetically regulated RNA-based stemness score (EREG.EXPss); **(F)** Epigenetically regulated DNA methylation-based stemness score (EREG-METHss).

**
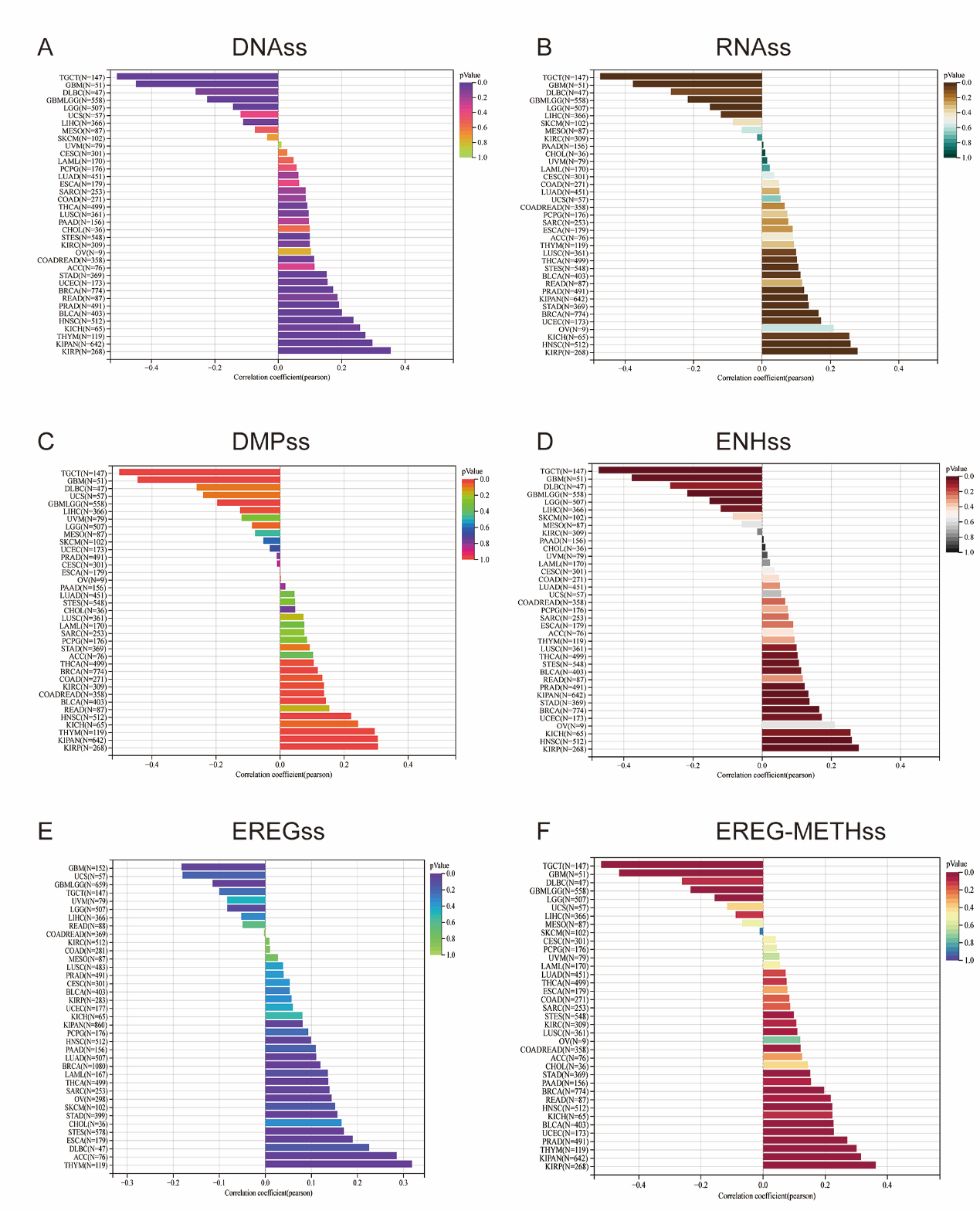
**
