# Supplementary material for: Pan-cancer analysis of PSCA that is associated with immune infiltration and affects patient prognosis
Source: PLoS One. 2024 Jun 25;19(6):e0298469. doi: 10.1371/journal.pone.0298469 (PMC11198779; doi:10.1371/journal.pone.0298469)

**Fig. S2 Effects of PSCA on genes related to RNA modification (m1A, m5C and m6A). (A)** Correlation between the expression of PSCA and RNA modification-related genes in pan-cancer; **(B–E)** PSCA is closely related to methylation modifications at multiple sites, including cg18270343 (r = 0.171, P < 0.001), cg10596483 (r = −0.157, P < 0.001), cg18270343 (r = −0.140, P = 0.007) and cg10596483 (r = −0.157, P < 0.001).


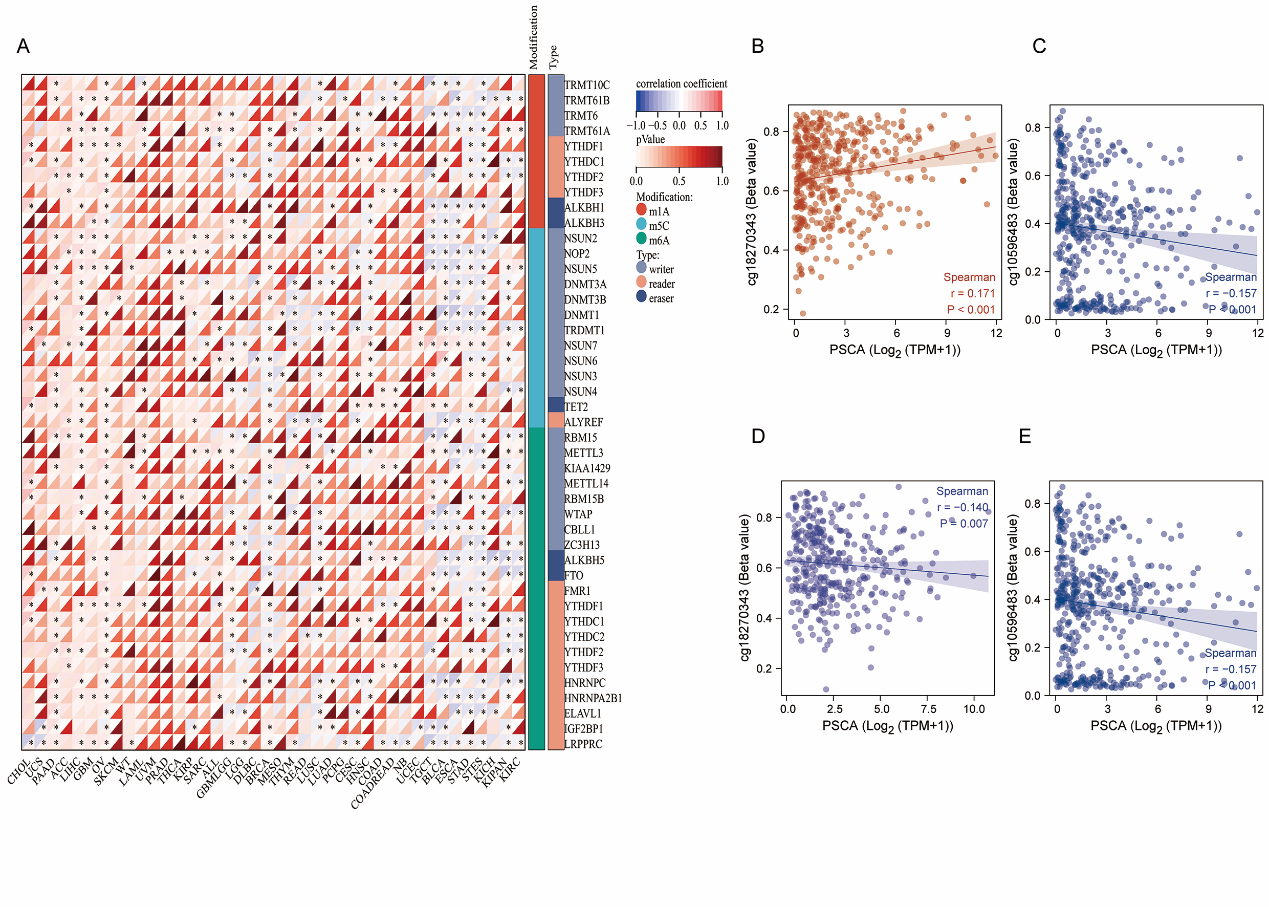

Supplement: S2 Fig — (A) Correlation between the expression of PSCA and RNA modification-related genes in pan-cancer; (B–E) PSCA is closely related to methylation modifications at multiple sites, including cg18270343 (r = 0.171, P < 0.001), cg10596483 (r = −0.157, P < 0.001), cg18270343 (r = −0.140, P = 0.007) and cg10596483 (r = −0.157, P < 0.001). (DOCX) [file pone.0298469.s002.docx]
