# Supplementary material for: Pan-cancer analysis of PSCA that is associated with immune infiltration and affects patient prognosis
Source: PLoS One. 2024 Jun 25;19(6):e0298469. doi: 10.1371/journal.pone.0298469 (PMC11198779; doi:10.1371/journal.pone.0298469)
Supplement: S3 Fig — Histograms demonstrate the relationship between PSCA expression and specific molecular subtypes of human cancers (A), stage (B), overall survival (C), immune cell subtypes (D), grade (E) and mutation difference between responders and non-responders (F) in pan-cancer. (DOCX) [file pone.0298469.s003.docx]

**Fig. S3** **Pan-cancer analysis of pathological features.** Histograms demonstrate the relationship between PSCA expression and specific molecular subtypes of human cancers **(A)**, stage **(B)**, overall survival**(C)**, immune cell subtypes **(D)**, grade **(E)** and mutation difference between responders and non-responders **(F)** in pan-cancer.

**
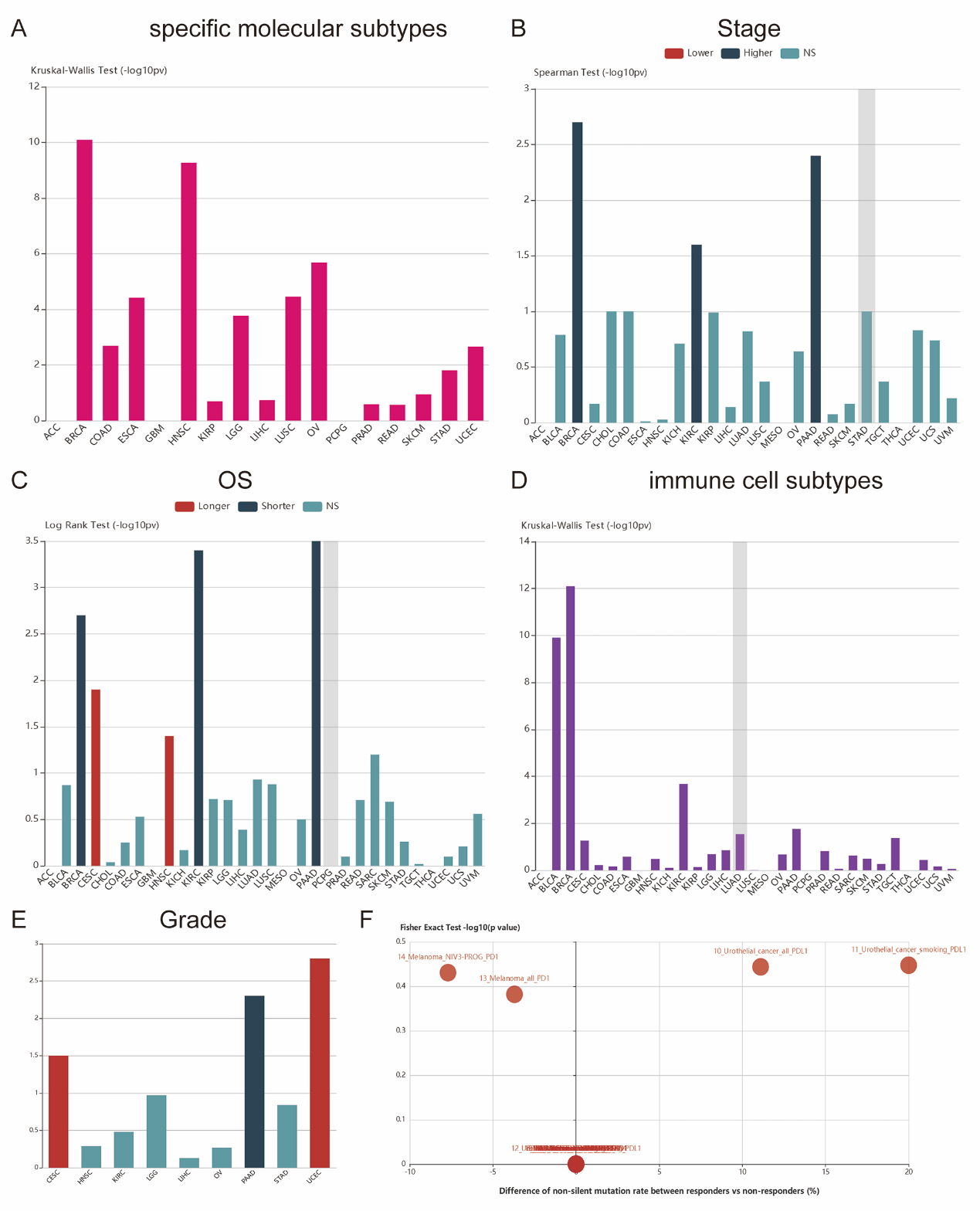
**
