# Supplementary material for: Pan-cancer analysis of PSCA that is associated with immune infiltration and affects patient prognosis
Source: PLoS One. 2024 Jun 25;19(6):e0298469. doi: 10.1371/journal.pone.0298469 (PMC11198779; doi:10.1371/journal.pone.0298469)

**Fig. S4 Genome-wide correlation analysis.** Genome-wide correlation between PSCA and other signatures in LUAD **(A),** GBM **(B),** STAD **(C),** OV **(D),** LUSC **(E)** and BRCA **(F)**.


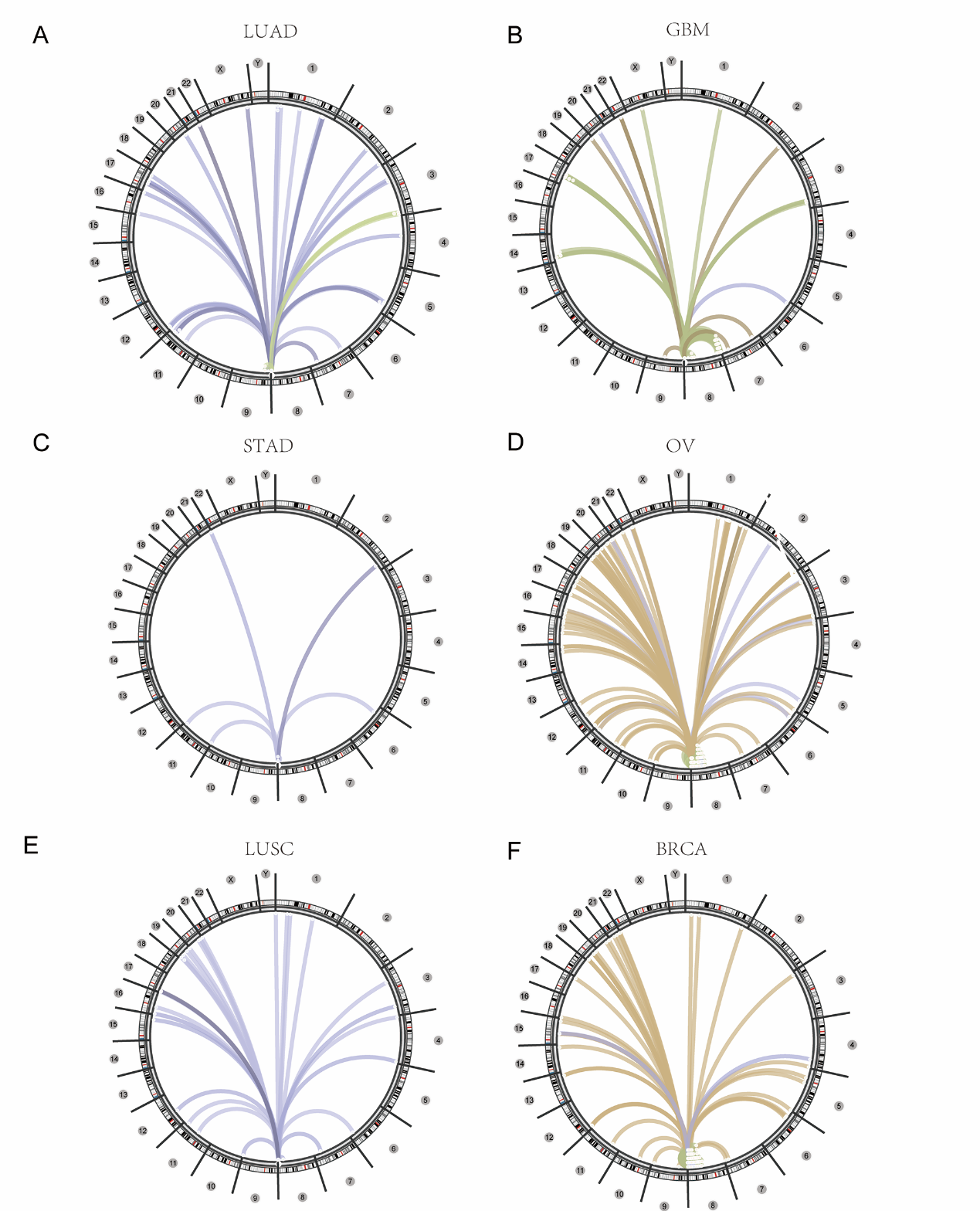

Supplement: S4 Fig — Genome-wide correlation between PSCA and other signatures in LUAD (A), GBM (B), STAD (C), OV (D), LUSC (E) and BRCA (F). (DOCX) [file pone.0298469.s004.docx]
