# Supplementary material for: Pan-cancer analysis of PSCA that is associated with immune infiltration and affects patient prognosis
Source: PLoS One. 2024 Jun 25;19(6):e0298469. doi: 10.1371/journal.pone.0298469 (PMC11198779; doi:10.1371/journal.pone.0298469)

**Fig. S5** **Validation of prognostic significance of PSCA in pan-cancer. (A)** OS in jacob-00182-UM dataset (cox.P = 0.004, HR = 1.44 [1.13–1.84]); **(B)** OS in GSE17536 dataset (cox.P = 0.02, HR = 0.07 [0.01–0.67]); **(C)** OS in ovarian cancer dataset GSE17260 (cox.P = 0.02, HR = 0.69 [0.51–0.94]); **(D)** OS in GSE8841 dataset (cox.P = 0.03, HR = 0.33 [0.12–0.89]); **(E)** DFS in GSE14333 dataset (cox.P = 0.001, HR = 1.34 [1.13–1.60]); **(F)** DFS in the eye cancer dataset GSE22138 (cox.P = 0.02, HR = 5.89 [1.35–25.66]).


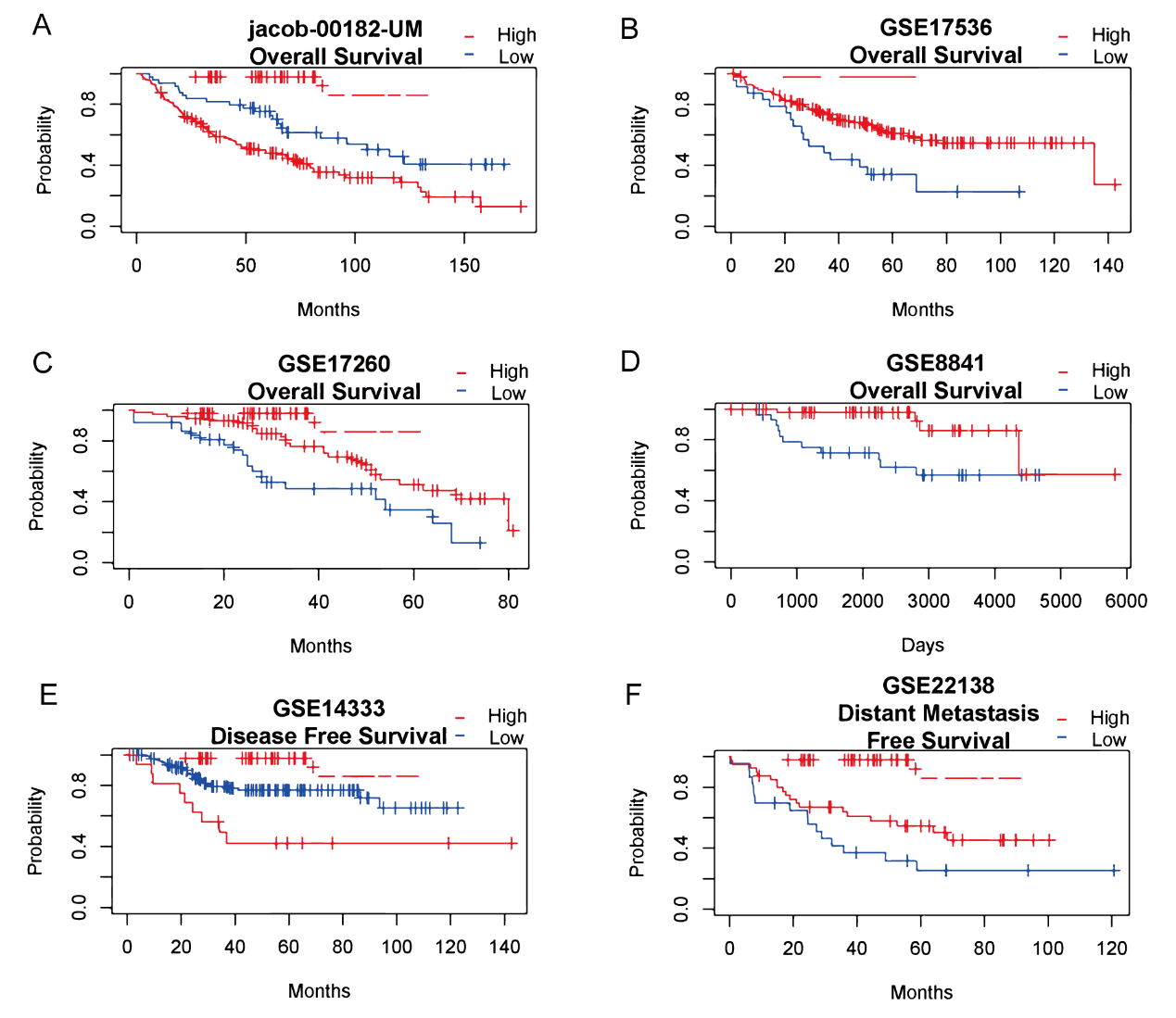

Supplement: S5 Fig — (A) OS in jacob-00182-UM dataset (cox.P = 0.004, HR = 1.44 [1.13–1.84]); (B) OS in GSE17536 dataset (cox.P = 0.02, HR = 0.07 [0.01–0.67]); (C) OS in ovarian cancer dataset GSE17260 (cox.P = 0.02, HR = 0.69 [0.51–0.94]); (D) OS in GSE8841 dataset (cox.P = 0.03, HR = 0.33 [0.12–0.89]); (E) DFS in GSE14333 dataset (cox.P = 0.001, HR = 1.34 [1.13–1.60]); (F) DFS in the eye cancer dataset GSE22138 (cox.P = 0.02, HR = 5.89 [1.35–25.66]). (DOCX) [file pone.0298469.s005.docx]
