# Supplementary material for: Pan-cancer analysis of PSCA that is associated with immune infiltration and affects patient prognosis
Source: PLoS One. 2024 Jun 25;19(6):e0298469. doi: 10.1371/journal.pone.0298469 (PMC11198779; doi:10.1371/journal.pone.0298469)

**Fig. S6** **Subgroup analysis based on clinicopathological characteristics in pan-cancer. (A–B)** Mutation pattern of PSCA in LUAD; **(C)** PSCA expression was significantly correlated with T stage in CESC and HNSC; **(D)** PSCA expression was significantly correlated with tumour stage in GBMLGG, BRCA and SKCM; **(E)** PSCA expression was significantly correlated with N stage in HNSC and SKCM; **(F)** The relationship between PSCA expression and tumour grade was not significant in pan-cancer; **(G)** PSCA expression was significantly correlated with M stage in COAD, THYM, READ and BLCA.


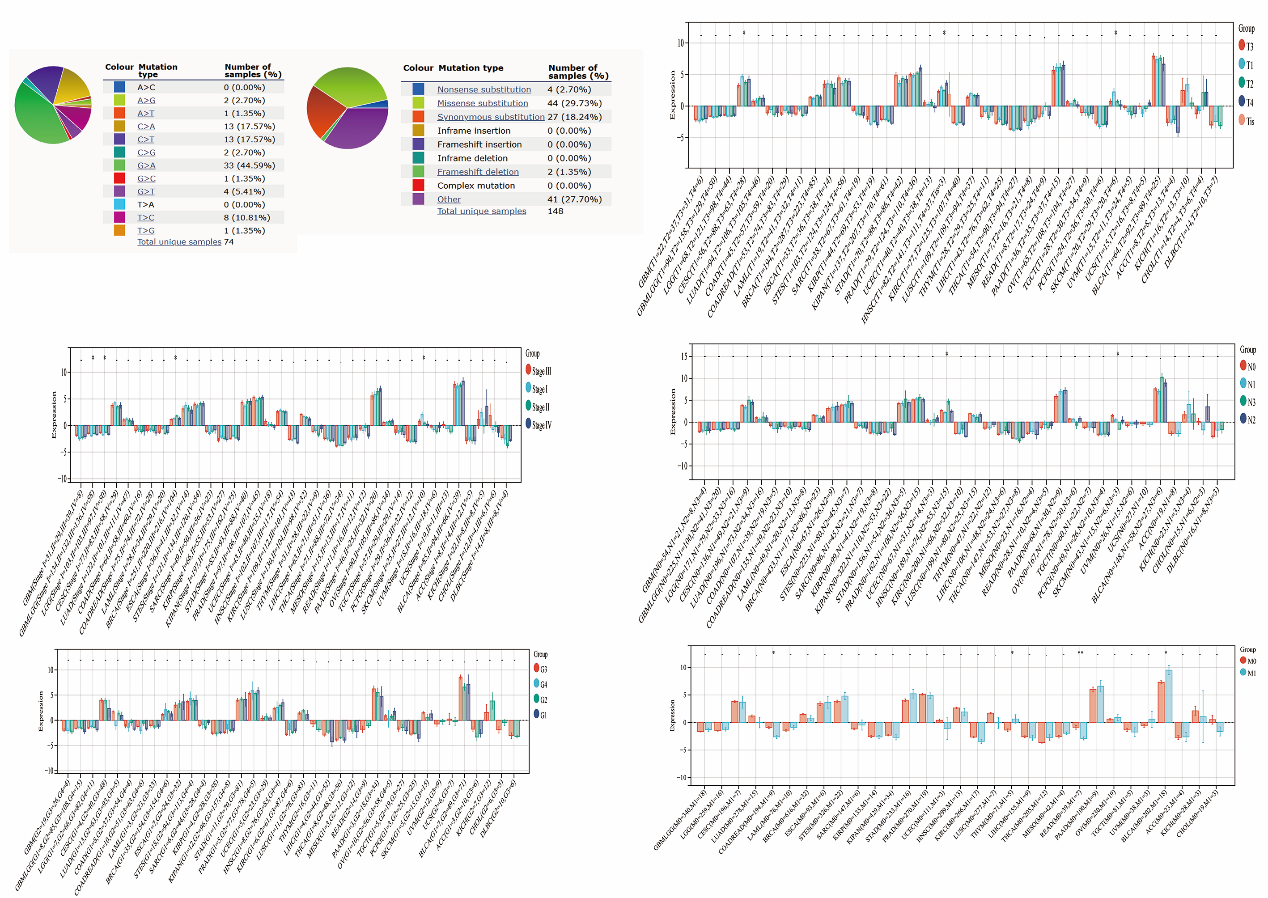

Supplement: S6 Fig — (A–B) Mutation pattern of PSCA in LUAD; (C) PSCA expression was significantly correlated with T stage in CESC and HNSC; (D) PSCA expression was significantly correlated with tumour stage in GBMLGG, BRCA and SKCM; (E) PSCA expression was significantly correlated with N stage in HNSC and SKCM; (F) The relationship between PSCA expression and tumour grade was not significant in pan-cancer; (G) PSCA expression was significantly correlated with M stage in COAD, THYM, READ and BLCA. (DOCX) [file pone.0298469.s006.docx]
