# Supplementary material for: Pan-cancer analysis of PSCA that is associated with immune infiltration and affects patient prognosis
Source: PLoS One. 2024 Jun 25;19(6):e0298469. doi: 10.1371/journal.pone.0298469 (PMC11198779; doi:10.1371/journal.pone.0298469)

**Fig. S7. PSCA affects immune regulation in tumours.** In each cancer sample, the gene expression values, CNA and methylation levels of total lymphocytes **(A),** immunosuppressor levels **(B),** immunostimulator levels **(C)** and MHC molecule levels **(D)** were calculated based on PSCA expression.
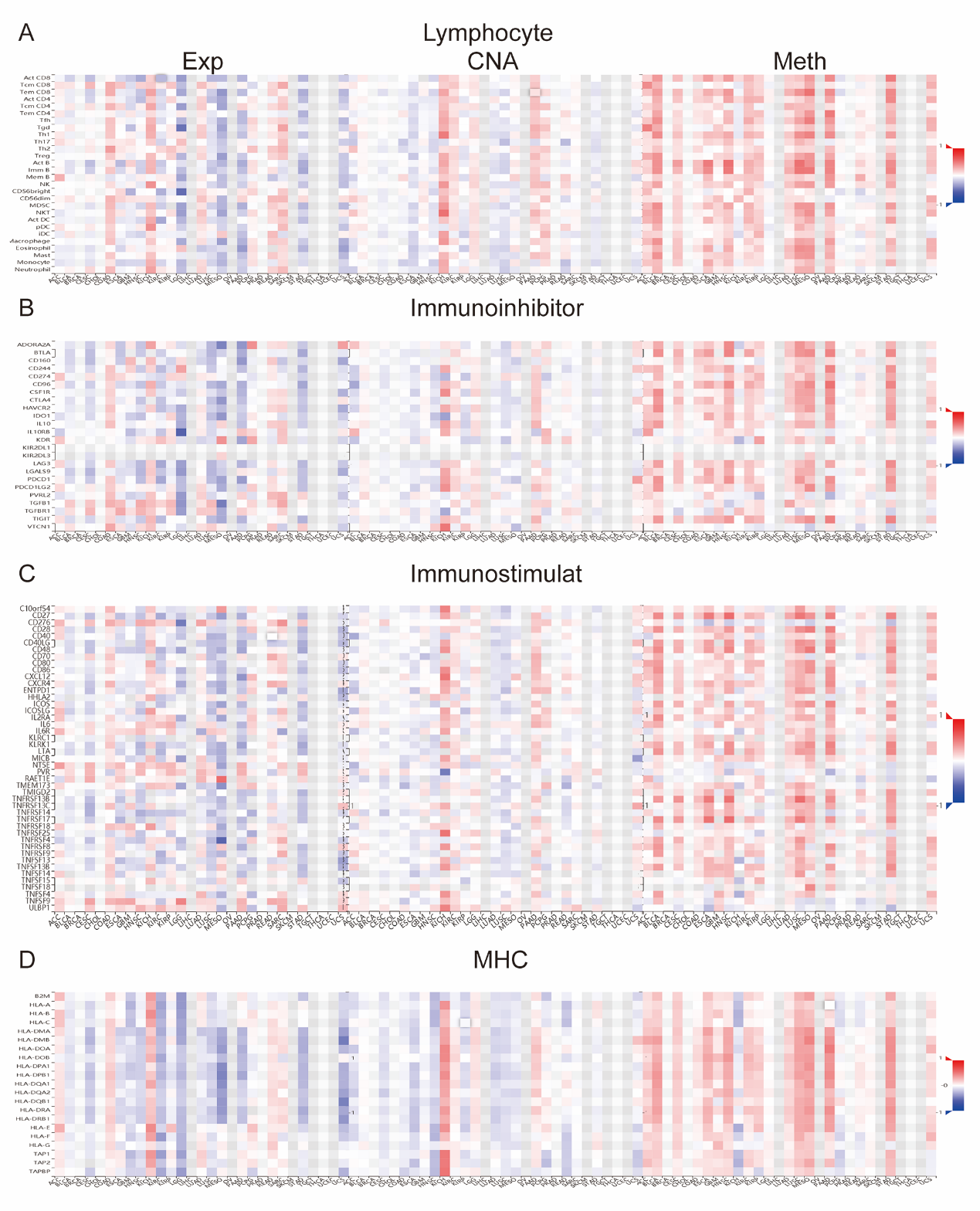

Supplement: S7 Fig — In each cancer sample, the gene expression values, CNA and methylation levels of total lymphocytes (A), immunosuppressor levels (B), immunostimulator levels (C) and MHC molecule levels (D) were calculated based on PSCA expression. (DOCX) [file pone.0298469.s007.docx]
