# Supplementary material for: Pan-cancer analysis of PSCA that is associated with immune infiltration and affects patient prognosis
Source: PLoS One. 2024 Jun 25;19(6):e0298469. doi: 10.1371/journal.pone.0298469 (PMC11198779; doi:10.1371/journal.pone.0298469)

**Fig. S8** **Role of PSCA in the prognosis of LUAD. (A–B)** Univariate and multivariate Cox regression analyses of T stage, N stage, M stage, treatment outcomes, pathological stage and PSCA expression in LUAD; **(C)** Nomogram integrating PSCA expression and clinical variables such as age, sex, pathological stage, TNM stage and treatment outcomes;**（D）**KM curves demonstrating the effects of PSCA expression of prognosis in LUAD;**（E）**Calibration curve validating the prognostic significance of clinicopathological characteristics of tumours.


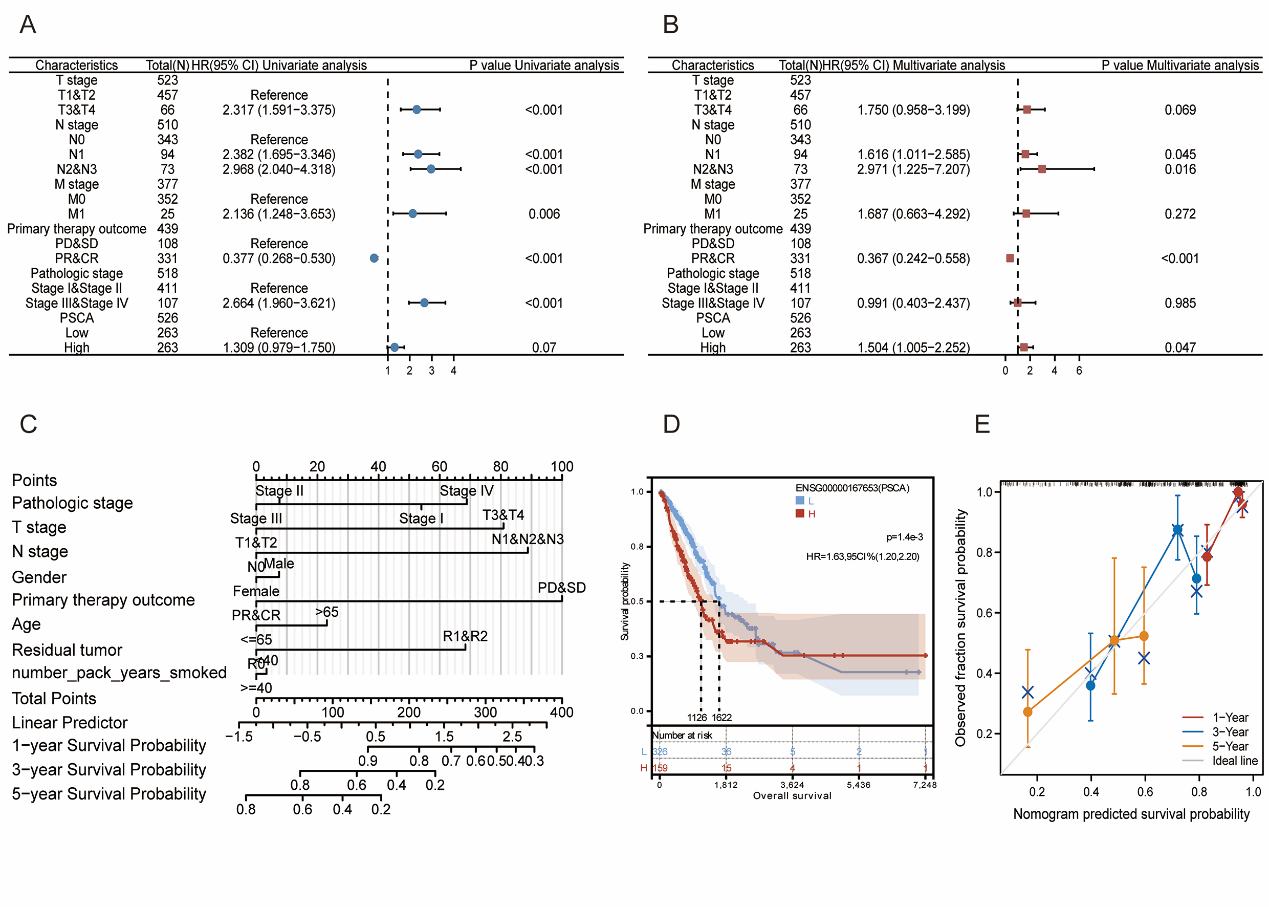

Supplement: S8 Fig — (A–B) Univariate and multivariate Cox regression analyses of T stage, N stage, M stage, treatment outcomes, pathological stage and PSCA expression in LUAD; (C) Nomogram integrating PSCA expression and clinical variables such as age, sex, pathological stage, TNM stage and treatment outcomes; (D) KM curves demonstrating the effects of PSCA expression of prognosis in LUAD; (E) Calibration curve validating the prognostic significance of clinicopathological characteristics of tumours. (DOCX) [file pone.0298469.s008.docx]
