# Supplementary material for: Pan-cancer analysis of PSCA that is associated with immune infiltration and affects patient prognosis
Source: PLoS One. 2024 Jun 25;19(6):e0298469. doi: 10.1371/journal.pone.0298469 (PMC11198779; doi:10.1371/journal.pone.0298469)

**Fig. S9 ROC curves were plotted to validate the diagnostic efficacy of PSCA. (A)** Discrimination efficacy of PSCA for LUAD**; (B–F)** PSCA had better discrimination efficacy for TNM stage, residual tumour and primary treatment outcomes.


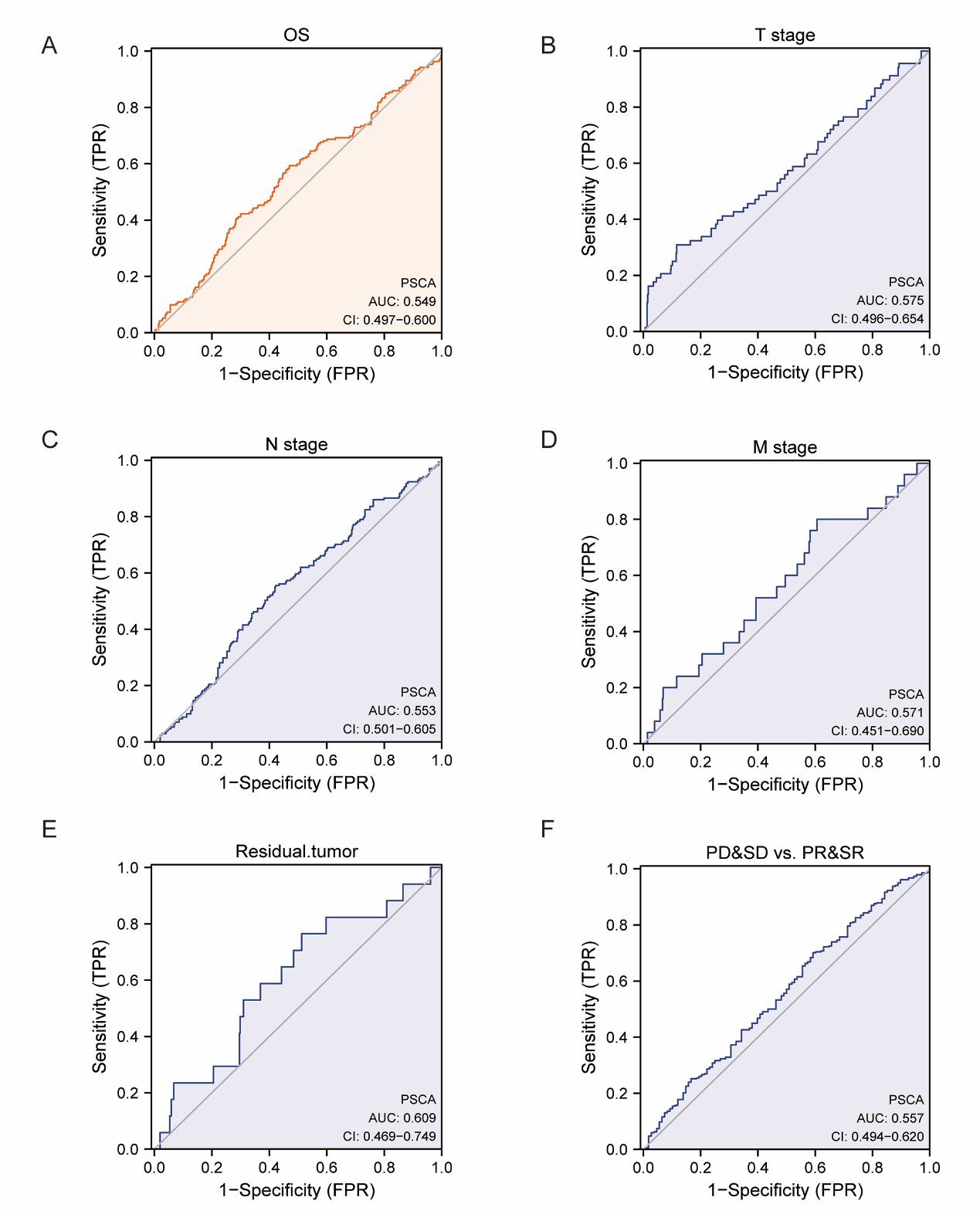

Supplement: S9 Fig — (A) Discrimination efficacy of PSCA for LUAD; (B–F) PSCA had better discrimination efficacy for TNM stage, residual tumour and primary treatment outcomes. (DOCX) [file pone.0298469.s009.docx]
