# Supplementary material for: Pan-cancer analysis of PSCA that is associated with immune infiltration and affects patient prognosis
Source: PLoS One. 2024 Jun 25;19(6):e0298469. doi: 10.1371/journal.pone.0298469 (PMC11198779; doi:10.1371/journal.pone.0298469)

**Fig. S10 Results of gene set enrichment analysis (GSEA) were summarised as enrichment maps. (A–F)** Pathway enrichment in the low-PSCA-expression group in the REACTOME database; **(G–L)** Pathway enrichment in the REACTOME, KEGG, PID, WP and BIOCARTA databases was statistically significant in the ‘c2.cp.KEGG.v7.1.symbols.gmt’ gene set.


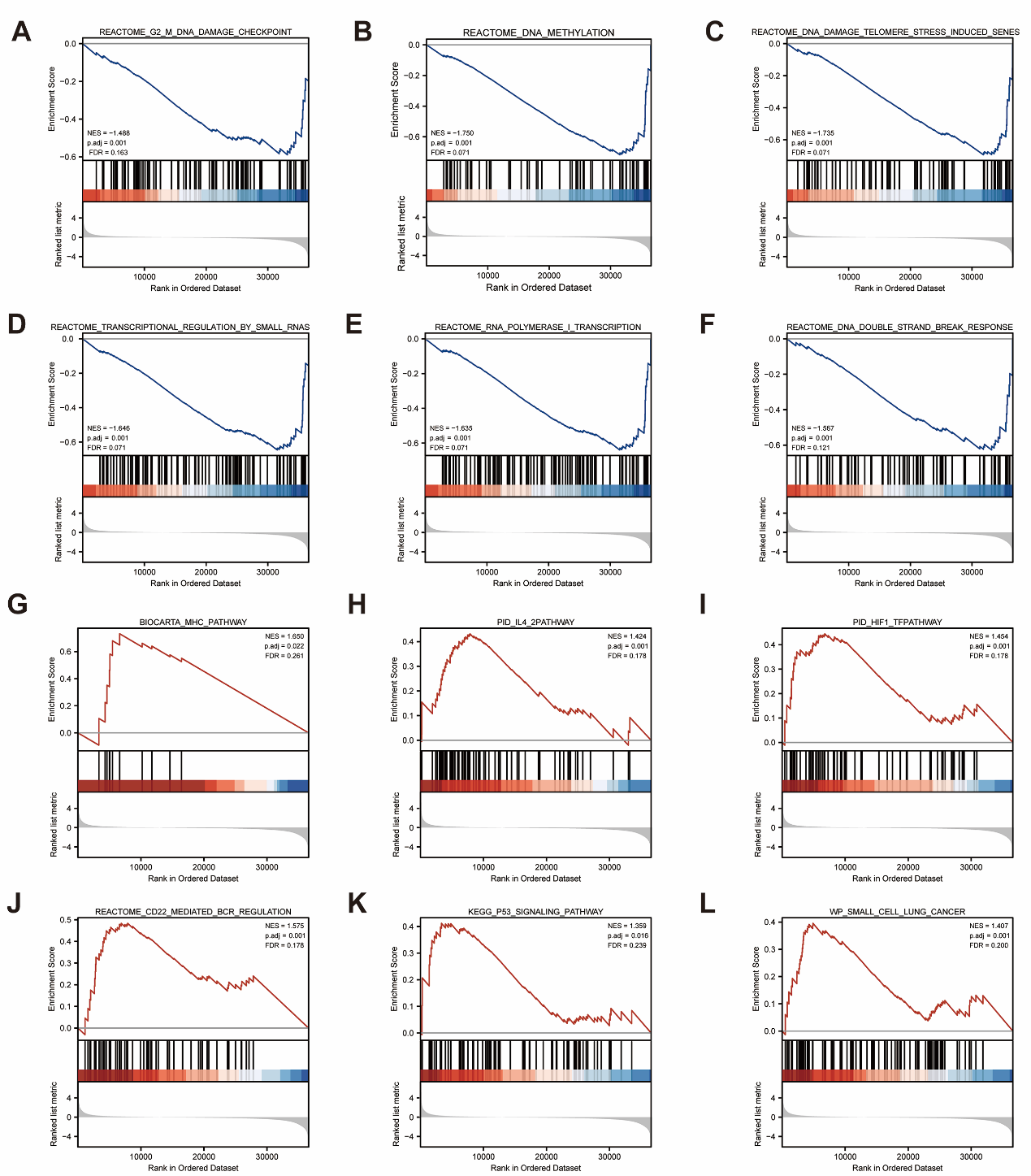

Supplement: S10 Fig — (A–F) Pathway enrichment in the low-PSCA-expression group in the REACTOME database; (G–L) Pathway enrichment in the REACTOME, KEGG, PID, WP and BIOCARTA databases was statistically significant in the ‘c2.cp.KEGG.v7.1.symbols.gmt’ gene set. (DOCX) [file pone.0298469.s010.docx]
