# Supplementary material for: Pan-cancer analysis of PSCA that is associated with immune infiltration and affects patient prognosis
Source: PLoS One. 2024 Jun 25;19(6):e0298469. doi: 10.1371/journal.pone.0298469 (PMC11198779; doi:10.1371/journal.pone.0298469)
Supplement: S1 Table — (DOCX) [file pone.0298469.s011.docx]

**S1 Table. Validation of survival prognosis analysis in multi-database**

| **DATASET** | **CANCER TYPE** |  | **ENDPOINT** | **P** | **HR [95% CI]** |
| --- | --- | --- | --- | --- | --- |
| GSE14333 | Colorectal cancer | | Disease Free Survival | 0.001012 | 1.34 [1.13 - 1.60] |
| GSE17536 | Colorectal cancer | | Disease Free Survival | 0.002559 | 1.90 [1.25 - 2.89] |
| jacob-00182-UM | Lung Adenocarcinoma |  | Overall Survival | 0.003506 | 1.44 [1.13 - 1.84] |
| GSE17260 | Ovarian cancer | | Overall Survival | 0.01712 | 0.69 [0.51 - 0.94] |
| GSE22138 | Eye cancer (Uveal melanoma) |  | Distant Metastasis Free Survival | 0.018098 | 5.89 [1.35 - 25.66] |
| GSE17536 | Colorectal cancer | | Overall Survival | 0.02033 | 0.07 [0.01 - 0.67] |
| GSE8841 | Ovarian cancer | | Overall Survival | 0.029064 | 0.33 [0.12 - 0.89] |
