# Supplementary material for: Pan-cancer analysis of PSCA that is associated with immune infiltration and affects patient prognosis
Source: PLoS One. 2024 Jun 25;19(6):e0298469. doi: 10.1371/journal.pone.0298469 (PMC11198779; doi:10.1371/journal.pone.0298469)
Supplement: S2 Table — (DOCX) [file pone.0298469.s012.docx]

**S2 Table. Univariate and multivariate regression analysis of PSCA for the prognosis of LUAD**

| **Characteristics** | **Total(N)** | **Univariate analysis** | |  | **Multivariate analysis** | |
| --- | --- | --- | --- | --- | --- | --- |
|  |  | **Hazard ratio (95% CI)** | **P value** |  | **Hazard ratio (95% CI)** | **P value** |
| T stage | 523 |  |  |  |  |  |
| T1&T2 | 457 | Reference |  |  |  |  |
| T3&T4 | 66 | 2.317 (1.591-3.375) | **<0.001** |  | 1.750 (0.958-3.199) | 0.069 |
| N stage | 510 |  |  |  |  |  |
| N0 | 343 | Reference |  |  |  |  |
| N1 | 94 | 2.382 (1.695-3.346) | **<0.001** |  | 1.616 (1.011-2.585) | **0.045** |
| N2&N3 | 73 | 2.968 (2.040-4.318) | **<0.001** |  | 2.971 (1.225-7.207) | **0.016** |
| M stage | 377 |  |  |  |  |  |
| M0 | 352 | Reference |  |  |  |  |
| M1 | 25 | 2.136 (1.248-3.653) | **0.006** |  | 1.687 (0.663-4.292) | 0.272 |
| Primary therapy outcome | 439 |  |  |  |  |  |
| PD&SD | 108 | Reference |  |  |  |  |
| PR&CR | 331 | 0.377 (0.268-0.530) | **<0.001** |  | 0.367 (0.242-0.558) | **<0.001** |
| Pathologic stage | 518 |  |  |  |  |  |
| Stage I&Stage II | 411 | Reference |  |  |  |  |
| Stage III&Stage IV | 107 | 2.664 (1.960-3.621) | **<0.001** |  | 0.991 (0.403-2.437) | 0.985 |
| PSCA | 526 |  |  |  |  |  |
| Low | 263 | Reference |  |  |  |  |
| High | 263 | 1.309 (0.979-1.750) | 0.070 |  | 1.504 (1.005-2.252) | **0.047** |
