# Supplementary material for: Pan-cancer analysis of PSCA that is associated with immune infiltration and affects patient prognosis
Source: PLoS One. 2024 Jun 25;19(6):e0298469. doi: 10.1371/journal.pone.0298469 (PMC11198779; doi:10.1371/journal.pone.0298469)
Supplement: S3 Table — (DOCX) [file pone.0298469.s013.docx]

**S3 Table. Logistic regression model of clinicopathological features based on PSCA expression levels.**

| Characteristics | Total(N) | Odds Ratio(OR) | P value |
| --- | --- | --- | --- |
| T stage (T2&T3&T4 vs. T1) | 532 | 1.248 (0.869-1.796) | 0.231 |
| N stage (N1&N2&N3 vs. N0) | 519 | 1.471 (1.019-2.130) | 0.040 |
| M stage (M1 vs. M0) | 386 | 1.667 (0.737-3.926) | 0.226 |
| Pathologic stage (Stage III&Stage IV vs. Stage I&Stage II) | 527 | 1.081 (0.710-1.648) | 0.718 |
| Primary therapy outcome (PR&CR vs. PD&SD) | 446 | 0.764 (0.494-1.180) | 0.226 |
| Residual tumor (R1&R2 vs. R0) | 372 | 3.343 (1.158-12.047) | 0.038 |
| Age (>65 vs. <=65) | 516 | 1.150 (0.814-1.625) | 0.428 |
| Smoker (Yes vs. No) | 521 | 0.965 (0.590-1.576) | 0.886 |
