# Supplementary material for: Pan-cancer analysis of PSCA that is associated with immune infiltration and affects patient prognosis
Source: PLoS One. 2024 Jun 25;19(6):e0298469. doi: 10.1371/journal.pone.0298469 (PMC11198779; doi:10.1371/journal.pone.0298469)
Supplement: S4 Table — (DOCX) [file pone.0298469.s014.docx]

**S4 Table. Canonical pathways MSigDB category (c2.cp.v5.2.symbols.gmt) was used for GSEA**

| Description | ES | NES | P | q |
| --- | --- | --- | --- | --- |
| BIOCARTA_MHC_PATHWAY | 0.73 | 1.65 | 0.02 | 0.26 |
| BIOCARTA_TFF_PATHWAY | 0.80 | 2.14 | 0.00 | 0.18 |
| KEGG_ASCORBATE_AND_ALDARATE_METABOLISM | 0.84 | 2.28 | 0.00 | 0.18 |
| KEGG_B_CELL_RECEPTOR_SIGNALING_PATHWAY | 0.44 | 1.48 | 0.02 | 0.26 |
| KEGG_CARDIAC_MUSCLE_CONTRACTION | 0.49 | 1.65 | 0.01 | 0.19 |
| KEGG_DRUG_METABOLISM_CYTOCHROME_P450 | 0.76 | 2.53 | 0.01 | 0.18 |
| KEGG_DRUG_METABOLISM_OTHER_ENZYMES | 0.77 | 2.38 | 0.01 | 0.18 |
| KEGG_GLIOMA | 0.42 | 1.39 | 0.01 | 0.18 |
| KEGG_GLYCOSAMINOGLYCAN_DEGRADATION | 0.68 | 1.73 | 0.01 | 0.20 |
| KEGG_LEUKOCYTE_TRANSENDOTHELIAL_MIGRATION | 0.40 | 1.46 | 0.02 | 0.24 |
| KEGG_LINOLEIC_ACID_METABOLISM | 0.60 | 1.71 | 0.02 | 0.20 |
| KEGG_LYSOSOME | 0.37 | 1.33 | 0.02 | 0.26 |
| KEGG_METABOLISM_OF_XENOBIOTICS_BY_CYTOCHROME_P450 | 0.76 | 2.52 | 0.01 | 0.18 |
| KEGG_OOCYTE_MEIOSIS | 0.40 | 1.43 | 0.02 | 0.24 |
| KEGG_OXIDATIVE_PHOSPHORYLATION | 0.46 | 1.65 | 0.02 | 0.24 |
| KEGG_P53_SIGNALING_PATHWAY | 0.41 | 1.36 | 0.02 | 0.24 |
| KEGG_PARKINSONS_DISEASE | 0.45 | 1.64 | 0.02 | 0.24 |
| KEGG_PENTOSE_AND_GLUCURONATE_INTERCONVERSIONS | 0.86 | 2.41 | 0.00 | 0.18 |
| KEGG_PENTOSE_PHOSPHATE_PATHWAY | 0.60 | 1.66 | 0.02 | 0.27 |
| KEGG_PORPHYRIN_AND_CHLOROPHYLL_METABOLISM | 0.82 | 2.44 | 0.01 | 0.18 |
| KEGG_RETINOL_METABOLISM | 0.75 | 2.49 | 0.01 | 0.18 |
| KEGG_STARCH_AND_SUCROSE_METABOLISM | 0.73 | 2.27 | 0.01 | 0.18 |
| KEGG_STEROID_HORMONE_BIOSYNTHESIS | 0.78 | 2.48 | 0.01 | 0.18 |
| KEGG_SYSTEMIC_LUPUS_ERYTHEMATOSUS | -0.56 | -1.46 | 0.00 | 0.12 |
| KEGG_TASTE_TRANSDUCTION | -0.65 | -1.53 | 0.00 | 0.12 |
| KEGG_VIBRIO_CHOLERAE_INFECTION | 0.49 | 1.55 | 0.01 | 0.18 |
| NABA_COLLAGENS | 0.49 | 1.49 | 0.01 | 0.20 |
| PID_A6B1_A6B4_INTEGRIN_PATHWAY | 0.61 | 1.88 | 0.01 | 0.18 |
| PID_ATF2_PATHWAY | 0.48 | 1.57 | 0.01 | 0.18 |
| PID_AVB3_INTEGRIN_PATHWAY | 0.49 | 1.64 | 0.01 | 0.19 |
| PID_DELTA_NP63_PATHWAY | 0.56 | 1.73 | 0.01 | 0.18 |
| PID_FRA_PATHWAY | 0.55 | 1.61 | 0.02 | 0.23 |
| PID_HIF1_TFPATHWAY | 0.44 | 1.45 | 0.01 | 0.18 |
| PID_HNF3A_PATHWAY | 0.50 | 1.52 | 0.01 | 0.20 |
| PID_IL4_2PATHWAY | 0.43 | 1.42 | 0.01 | 0.18 |
| PID_INTEGRIN1_PATHWAY | 0.50 | 1.65 | 0.01 | 0.18 |
| PID_INTEGRIN3_PATHWAY | 0.49 | 1.49 | 0.01 | 0.20 |
| PID_INTEGRIN4_PATHWAY | 0.77 | 1.73 | 0.02 | 0.25 |
| PID_MYC_REPRESS_PATHWAY | 0.47 | 1.52 | 0.01 | 0.18 |
| PID_P75_NTR_PATHWAY | 0.46 | 1.51 | 0.02 | 0.24 |
| PID_SYNDECAN_1_PATHWAY | 0.63 | 1.95 | 0.01 | 0.18 |
| PID_TAP63_PATHWAY | 0.62 | 1.95 | 0.01 | 0.18 |
| PID_UPA_UPAR_PATHWAY | 0.51 | 1.53 | 0.01 | 0.20 |
| PID_WNT_SIGNALING_PATHWAY | 0.60 | 1.64 | 0.02 | 0.27 |
| REACTOME_ACTIVATED_PKN1_STIMULATES_TRANSCRIPTION_OF_AR_ANDROGEN_RECEPTOR_REGULATED_GENES_KLK2_AND_KLK3 | -0.71 | -1.74 | 0.00 | 0.07 |
| REACTOME_ACTIVATION_OF_ANTERIOR_HOX_GENES_IN_HINDBRAIN_DEVELOPMENT_DURING_EARLY_EMBRYOGENESIS | -0.59 | -1.54 | 0.00 | 0.07 |
| REACTOME_ACTIVATION_OF_MATRIX_METALLOPROTEINASES | 0.75 | 2.15 | 0.01 | 0.18 |
| REACTOME_AFLATOXIN_ACTIVATION_AND_DETOXIFICATION | 0.78 | 1.95 | 0.00 | 0.18 |
| REACTOME_AMYLOID_FIBER_FORMATION | -0.57 | -1.46 | 0.01 | 0.18 |
| REACTOME_ANCHORING_FIBRIL_FORMATION | 0.76 | 1.78 | 0.02 | 0.21 |
| REACTOME_ANDROGEN_BIOSYNTHESIS | 0.76 | 1.71 | 0.03 | 0.28 |
| REACTOME_ANTIGEN_PROCESSING_CROSS_PRESENTATION | 0.43 | 1.55 | 0.01 | 0.20 |
| REACTOME_ANTIMICROBIAL_PEPTIDES | 0.56 | 1.89 | 0.01 | 0.20 |
| REACTOME_APOPTOSIS_INDUCED_DNA_FRAGMENTATION | -0.81 | -1.58 | 0.01 | 0.18 |
| REACTOME_ARACHIDONIC_ACID_METABOLISM | 0.46 | 1.51 | 0.01 | 0.18 |
| REACTOME_ASSEMBLY_OF_COLLAGEN_FIBRILS_AND_OTHER_MULTIMERIC_STRUCTURES | 0.71 | 2.30 | 0.01 | 0.18 |
| REACTOME_ATTENUATION_PHASE | 0.61 | 1.71 | 0.01 | 0.20 |
| REACTOME_AUF1_HNRNP_D0_BINDS_AND_DESTABILIZES_MRNA | 0.50 | 1.59 | 0.01 | 0.18 |
| REACTOME_B_WICH_COMPLEX_POSITIVELY_REGULATES_RRNA_EXPRESSION | -0.67 | -1.70 | 0.00 | 0.07 |
| REACTOME_BASE_EXCISION_REPAIR | -0.66 | -1.67 | 0.00 | 0.07 |
| REACTOME_BASE_EXCISION_REPAIR_AP_SITE_FORMATION | -0.72 | -1.74 | 0.00 | 0.07 |
| REACTOME_CARBOXYTERMINAL_POST_TRANSLATIONAL_MODIFICATIONS_OF_TUBULIN | 0.62 | 1.88 | 0.01 | 0.18 |
| REACTOME_CD22_MEDIATED_BCR_REGULATION | 0.48 | 1.58 | 0.01 | 0.18 |
| REACTOME_CELL_JUNCTION_ORGANIZATION | 0.50 | 1.69 | 0.01 | 0.20 |
| REACTOME_CELLULAR_SENESCENCE | -0.54 | -1.44 | 0.00 | 0.12 |
| REACTOME_CHEMOKINE_RECEPTORS_BIND_CHEMOKINES | 0.54 | 1.73 | 0.01 | 0.18 |
| REACTOME_CHROMOSOME_MAINTENANCE | -0.55 | -1.44 | 0.00 | 0.16 |
| REACTOME_COLLAGEN_BIOSYNTHESIS_AND_MODIFYING_ENZYMES | 0.50 | 1.64 | 0.01 | 0.18 |
| REACTOME_COLLAGEN_CHAIN_TRIMERIZATION | 0.49 | 1.49 | 0.01 | 0.20 |
| REACTOME_COLLAGEN_DEGRADATION | 0.62 | 2.04 | 0.01 | 0.18 |
| REACTOME_COLLAGEN_FORMATION | 0.60 | 2.03 | 0.01 | 0.20 |
| REACTOME_CONDENSATION_OF_PROPHASE_CHROMOSOMES | -0.70 | -1.73 | 0.00 | 0.07 |
| REACTOME_COPI_INDEPENDENT_GOLGI_TO_ER_RETROGRADE_TRAFFIC | 0.45 | 1.41 | 0.01 | 0.18 |
| REACTOME_CREATION_OF_C4_AND_C2_ACTIVATORS | 0.48 | 1.60 | 0.01 | 0.18 |
| REACTOME_CYP2E1_REACTIONS | 0.83 | 1.87 | 0.01 | 0.18 |
| REACTOME_CYTOCHROME_P450_ARRANGED_BY_SUBSTRATE_TYPE | 0.61 | 2.02 | 0.01 | 0.18 |
| REACTOME_CYTOSOLIC_SULFONATION_OF_SMALL_MOLECULES | 0.68 | 1.80 | 0.01 | 0.20 |
| REACTOME_DECTIN_1_MEDIATED_NONCANONICAL_NF_KB_SIGNALING | 0.45 | 1.45 | 0.01 | 0.18 |
| REACTOME_DECTIN_2_FAMILY | 0.84 | 2.28 | 0.00 | 0.18 |
| REACTOME_DEFECTIVE_C1GALT1C1_CAUSES_TN_POLYAGGLUTINATION_SYNDROME_TNPS_ | 0.90 | 2.19 | 0.00 | 0.18 |
| REACTOME_DEFECTIVE_CFTR_CAUSES_CYSTIC_FIBROSIS | 0.47 | 1.52 | 0.01 | 0.18 |
| REACTOME_DEFECTIVE_GALNT3_CAUSES_FAMILIAL_HYPERPHOSPHATEMIC_TUMORAL_CALCINOSIS_HFTC_ | 0.91 | 2.17 | 0.00 | 0.18 |
| REACTOME_DEGRADATION_OF_DVL | 0.49 | 1.57 | 0.01 | 0.18 |
| REACTOME_DEGRADATION_OF_GLI1_BY_THE_PROTEASOME | 0.42 | 1.38 | 0.02 | 0.22 |
| REACTOME_DEPOSITION_OF_NEW_CENPA_CONTAINING_NUCLEOSOMES_AT_THE_CENTROMERE | -0.68 | -1.69 | 0.00 | 0.07 |
| REACTOME_DISEASES_ASSOCIATED_WITH_O_GLYCOSYLATION_OF_PROTEINS | 0.58 | 1.91 | 0.01 | 0.18 |
| REACTOME_DNA_DAMAGE_TELOMERE_STRESS_INDUCED_SENESCENCE | -0.69 | -1.74 | 0.00 | 0.07 |
| REACTOME_DNA_DOUBLE_STRAND_BREAK_RESPONSE | -0.63 | -1.57 | 0.00 | 0.12 |
| REACTOME_DNA_METHYLATION | -0.72 | -1.75 | 0.00 | 0.07 |
| REACTOME_EPIGENETIC_REGULATION_OF_GENE_EXPRESSION | -0.58 | -1.54 | 0.00 | 0.07 |
| REACTOME_ERCC6_CSB_AND_EHMT2_G9A_POSITIVELY_REGULATE_RRNA_EXPRESSION | -0.70 | -1.75 | 0.00 | 0.07 |
| REACTOME_FCGR_ACTIVATION | 0.41 | 1.37 | 0.02 | 0.24 |
| REACTOME_FORMATION_OF_SENESCENCE_ASSOCIATED_HETEROCHROMATIN_FOCI_SAHF_ | -0.74 | -1.51 | 0.02 | 0.24 |
| REACTOME_FORMATION_OF_THE_BETA_CATENIN_TCF_TRANSACTIVATING_COMPLEX | -0.65 | -1.64 | 0.00 | 0.07 |
| REACTOME_FORMATION_OF_THE_CORNIFIED_ENVELOPE | 0.74 | 2.70 | 0.02 | 0.27 |
| REACTOME_G2_M_DNA_DAMAGE_CHECKPOINT | -0.59 | -1.49 | 0.00 | 0.16 |
| REACTOME_GAP_JUNCTION_ASSEMBLY | 0.71 | 2.11 | 0.01 | 0.18 |
| REACTOME_GAP_JUNCTION_TRAFFICKING_AND_REGULATION | 0.68 | 2.11 | 0.01 | 0.18 |
| REACTOME_GENE_SILENCING_BY_RNA | -0.54 | -1.41 | 0.01 | 0.18 |
| REACTOME_GLUCURONIDATION | 0.76 | 2.08 | 0.01 | 0.18 |
| REACTOME_GLUTATHIONE_CONJUGATION | 0.58 | 1.69 | 0.01 | 0.18 |
| REACTOME_GLYCOSAMINOGLYCAN_METABOLISM | 0.42 | 1.54 | 0.02 | 0.27 |
| REACTOME_HATS_ACETYLATE_HISTONES | -0.61 | -1.61 | 0.00 | 0.07 |
| REACTOME_HCMV_EARLY_EVENTS | -0.58 | -1.51 | 0.00 | 0.07 |
| REACTOME_HCMV_INFECTION | -0.54 | -1.43 | 0.00 | 0.16 |
| REACTOME_HCMV_LATE_EVENTS | -0.63 | -1.62 | 0.00 | 0.07 |
| REACTOME_HDACS_DEACETYLATE_HISTONES | -0.69 | -1.74 | 0.00 | 0.07 |
| REACTOME_HDMS_DEMETHYLATE_HISTONES | -0.71 | -1.66 | 0.00 | 0.07 |
| REACTOME_HSF1_ACTIVATION | 0.60 | 1.73 | 0.01 | 0.18 |
| REACTOME_HSP90_CHAPERONE_CYCLE_FOR_STEROID_HORMONE_RECEPTORS_SHR_ | 0.54 | 1.73 | 0.01 | 0.18 |
| REACTOME_INHIBITION_OF_DNA_RECOMBINATION_AT_TELOMERE | -0.71 | -1.74 | 0.00 | 0.07 |
| REACTOME_INITIAL_TRIGGERING_OF_COMPLEMENT | 0.49 | 1.66 | 0.01 | 0.19 |
| REACTOME_INTEGRIN_CELL_SURFACE_INTERACTIONS | 0.47 | 1.59 | 0.01 | 0.20 |
| REACTOME_INTERFERON_ALPHA_BETA_SIGNALING | 0.49 | 1.64 | 0.01 | 0.18 |
| REACTOME_INTERFERON_GAMMA_SIGNALING | 0.58 | 2.00 | 0.01 | 0.20 |
| REACTOME_INTERLEUKIN_20_FAMILY_SIGNALING | 0.62 | 1.68 | 0.01 | 0.20 |
| REACTOME_INTRAFLAGELLAR_TRANSPORT | 0.52 | 1.66 | 0.01 | 0.18 |
| REACTOME_LAMININ_INTERACTIONS | 0.65 | 1.82 | 0.01 | 0.18 |
| REACTOME_MAPK6_MAPK4_SIGNALING | 0.47 | 1.62 | 0.01 | 0.20 |
| REACTOME_MEIOSIS | -0.55 | -1.43 | 0.01 | 0.19 |
| REACTOME_MEIOTIC_RECOMBINATION | -0.65 | -1.64 | 0.00 | 0.07 |
| REACTOME_MEIOTIC_SYNAPSIS | -0.60 | -1.49 | 0.01 | 0.18 |
| REACTOME_MET_PROMOTES_CELL_MOTILITY | 0.50 | 1.49 | 0.01 | 0.20 |
| REACTOME_METABOLISM_OF_STEROID_HORMONES | 0.63 | 1.83 | 0.01 | 0.18 |
| REACTOME_METABOLISM_OF_WATER_SOLUBLE_VITAMINS_AND_COFACTORS | 0.42 | 1.51 | 0.02 | 0.27 |
| REACTOME_METALLOTHIONEINS_BIND_METALS | 0.80 | 1.80 | 0.01 | 0.18 |
| REACTOME_MITOTIC_PROPHASE | -0.59 | -1.54 | 0.00 | 0.07 |
| REACTOME_NEGATIVE_EPIGENETIC_REGULATION_OF_RRNA_EXPRESSION | -0.65 | -1.65 | 0.00 | 0.07 |
| REACTOME_NEGATIVE_REGULATION_OF_NOTCH4_SIGNALING | 0.42 | 1.33 | 0.02 | 0.27 |
| REACTOME_NON_INTEGRIN_MEMBRANE_ECM_INTERACTIONS | 0.48 | 1.57 | 0.01 | 0.18 |
| REACTOME_NONHOMOLOGOUS_END_JOINING_NHEJ_ | -0.66 | -1.62 | 0.00 | 0.07 |
| REACTOME_O_LINKED_GLYCOSYLATION | 0.51 | 1.86 | 0.02 | 0.23 |
| REACTOME_O_LINKED_GLYCOSYLATION_OF_MUCINS | 0.67 | 2.19 | 0.01 | 0.18 |
| REACTOME_ORC1_REMOVAL_FROM_CHROMATIN | 0.41 | 1.38 | 0.02 | 0.24 |
| REACTOME_OXIDATIVE_STRESS_INDUCED_SENESCENCE | -0.59 | -1.53 | 0.00 | 0.07 |
| REACTOME_PHASE_I_FUNCTIONALIZATION_OF_COMPOUNDS | 0.59 | 2.09 | 0.02 | 0.20 |
| REACTOME_PHASE_II_CONJUGATION_OF_COMPOUNDS | 0.60 | 2.15 | 0.02 | 0.21 |
| REACTOME_PKMTS_METHYLATE_HISTONE_LYSINES | -0.59 | -1.45 | 0.02 | 0.20 |
| REACTOME_POSITIVE_EPIGENETIC_REGULATION_OF_RRNA_EXPRESSION | -0.65 | -1.65 | 0.00 | 0.07 |
| REACTOME_POST_TRANSLATIONAL_MODIFICATION_SYNTHESIS_OF_GPI_ANCHORED_PROTEINS | 0.68 | 2.29 | 0.01 | 0.20 |
| REACTOME_POU5F1_OCT4_SOX2_NANOG_REPRESS_GENES_RELATED_TO_DIFFERENTIATION | 0.84 | 1.86 | 0.01 | 0.20 |
| REACTOME_PRC2_METHYLATES_HISTONES_AND_DNA | -0.71 | -1.75 | 0.00 | 0.07 |
| REACTOME_PRE_NOTCH_EXPRESSION_AND_PROCESSING | -0.53 | -1.37 | 0.02 | 0.26 |
| REACTOME_PROCESSING_OF_DNA_DOUBLE_STRAND_BREAK_ENDS | -0.59 | -1.48 | 0.00 | 0.18 |
| REACTOME_RECOGNITION_AND_ASSOCIATION_OF_DNA_GLYCOSYLASE_WITH_SITE_CONTAINING_AN_AFFECTED_PURINE | -0.73 | -1.75 | 0.00 | 0.07 |
| REACTOME_REGULATION_OF_RUNX3_EXPRESSION_AND_ACTIVITY | 0.46 | 1.46 | 0.01 | 0.18 |
| REACTOME_RESPONSE_TO_METAL_IONS | 0.79 | 1.80 | 0.01 | 0.18 |
| REACTOME_RHO_GTPASES_ACTIVATE_NADPH_OXIDASES | 0.63 | 1.68 | 0.02 | 0.24 |
| REACTOME_RHO_GTPASES_ACTIVATE_PKNS | -0.63 | -1.60 | 0.00 | 0.07 |
| REACTOME_RMTS_METHYLATE_HISTONE_ARGININES | -0.69 | -1.71 | 0.00 | 0.07 |
| REACTOME_RNA_POLYMERASE_I_PROMOTER_ESCAPE | -0.68 | -1.72 | 0.00 | 0.07 |
| REACTOME_RNA_POLYMERASE_I_TRANSCRIPTION | -0.64 | -1.64 | 0.00 | 0.07 |
| REACTOME_RNA_POLYMERASE_II_TRANSCRIBES_SNRNA_GENES | -0.59 | -1.47 | 0.01 | 0.18 |
| REACTOME_RUNX1_REGULATES_GENES_INVOLVED_IN_MEGAKARYOCYTE_DIFFERENTIATION_AND_PLATELET_FUNCTION | -0.62 | -1.55 | 0.00 | 0.07 |
| REACTOME_RUNX1_REGULATES_TRANSCRIPTION_OF_GENES_INVOLVED_IN_DIFFERENTIATION_OF_HSCS | -0.58 | -1.52 | 0.00 | 0.07 |
| REACTOME_SCF_SKP2_MEDIATED_DEGRADATION_OF_P27_P21 | 0.47 | 1.52 | 0.01 | 0.18 |
| REACTOME_SENESCENCE_ASSOCIATED_SECRETORY_PHENOTYPE_SASP_ | -0.63 | -1.61 | 0.00 | 0.07 |
| REACTOME_SIGNALING_BY_RETINOIC_ACID | 0.49 | 1.48 | 0.02 | 0.24 |
| REACTOME_SIRT1_NEGATIVELY_REGULATES_RRNA_EXPRESSION | -0.71 | -1.74 | 0.00 | 0.07 |
| REACTOME_SPHINGOLIPID_DE_NOVO_BIOSYNTHESIS | 0.55 | 1.68 | 0.01 | 0.18 |
| REACTOME_SPHINGOLIPID_METABOLISM | 0.44 | 1.52 | 0.01 | 0.20 |
| REACTOME_SUMOYLATION_OF_CHROMATIN_ORGANIZATION_PROTEINS | -0.58 | -1.43 | 0.01 | 0.20 |
| REACTOME_TELOMERE_MAINTENANCE | -0.60 | -1.54 | 0.00 | 0.12 |
| REACTOME_TERMINATION_OF_O_GLYCAN_BIOSYNTHESIS | 0.86 | 2.27 | 0.00 | 0.18 |
| REACTOME_THE_ROLE_OF_GTSE1_IN_G2_M_PROGRESSION_AFTER_G2_CHECKPOINT | 0.49 | 1.67 | 0.01 | 0.19 |
| REACTOME_THYROXINE_BIOSYNTHESIS | 0.87 | 1.93 | 0.00 | 0.17 |
| REACTOME_TNFR2_NON_CANONICAL_NF_KB_PATHWAY | 0.41 | 1.49 | 0.01 | 0.20 |
| REACTOME_TRANSCRIPTIONAL_REGULATION_BY_SMALL_RNAS | -0.65 | -1.65 | 0.00 | 0.07 |
| REACTOME_TRANSCRIPTIONAL_REGULATION_BY_THE_AP_2_TFAP2_FAMILY_OF_TRANSCRIPTION_FACTORS | 0.53 | 1.56 | 0.02 | 0.24 |
| REACTOME_TRANSCRIPTIONAL_REGULATION_OF_GRANULOPOIESIS | -0.65 | -1.65 | 0.00 | 0.07 |
| REACTOME_TRANSPORT_OF_CONNEXONS_TO_THE_PLASMA_MEMBRANE | 0.66 | 1.68 | 0.01 | 0.20 |
| REACTOME_TYPE_I_HEMIDESMOSOME_ASSEMBLY | 0.92 | 2.07 | 0.00 | 0.17 |
| REACTOME_XENOBIOTICS | 0.77 | 2.05 | 0.01 | 0.18 |
| WP_22Q112_DELETION_SYNDROME | 0.41 | 1.46 | 0.01 | 0.20 |
| WP_3Q29_COPY_NUMBER_VARIATION_SYNDROME | 0.52 | 1.76 | 0.01 | 0.19 |
| WP_AGERAGE_PATHWAY | 0.44 | 1.43 | 0.01 | 0.18 |
| WP_ARYL_HYDROCARBON_RECEPTOR_PATHWAY | 0.67 | 2.06 | 0.01 | 0.18 |
| WP_CARDIAC_PROGENITOR_DIFFERENTIATION | 0.53 | 1.68 | 0.01 | 0.18 |
| WP_CODEINE_AND_MORPHINE_METABOLISM | 0.84 | 1.95 | 0.00 | 0.18 |
| WP_CONSTITUTIVE_ANDROSTANE_RECEPTOR_PATHWAY | 0.65 | 1.85 | 0.01 | 0.18 |
| WP_CORI_CYCLE | 0.77 | 1.88 | 0.01 | 0.18 |
| WP_ESTROGEN_METABOLISM | 0.74 | 1.83 | 0.01 | 0.18 |
| WP_GASTRIN_SIGNALING_PATHWAY | 0.54 | 1.94 | 0.02 | 0.24 |
| WP_GLUCURONIDATION | 0.85 | 2.31 | 0.00 | 0.18 |
| WP_HAIR_FOLLICLE_DEVELOPMENT_CYTODIFFERENTIATION_PART_3_OF_3 | 0.56 | 1.95 | 0.01 | 0.20 |
| WP_HISTONE_MODIFICATIONS | -0.63 | -1.55 | 0.00 | 0.18 |
| WP_INTEGRINMEDIATED_CELL_ADHESION | 0.45 | 1.59 | 0.02 | 0.20 |
| WP_INTRAFLAGELLAR_TRANSPORT_PROTEINS_BINDING_TO_DYNEIN | 0.62 | 1.70 | 0.02 | 0.24 |
| WP_IRINOTECAN_PATHWAY | 0.83 | 1.89 | 0.00 | 0.18 |
| WP_NUCLEAR_RECEPTORS_IN_LIPID_METABOLISM_AND_TOXICITY | 0.58 | 1.68 | 0.01 | 0.18 |
| WP_OXIDATION_BY_CYTOCHROME_P450 | 0.61 | 1.99 | 0.01 | 0.18 |
| WP_OXIDATIVE_PHOSPHORYLATION | 0.49 | 1.52 | 0.01 | 0.18 |
| WP_PARKINUBIQUITIN_PROTEASOMAL_SYSTEM_PATHWAY | 0.41 | 1.38 | 0.02 | 0.24 |
| WP_PREGNANE_X_RECEPTOR_PATHWAY | 0.70 | 2.00 | 0.01 | 0.18 |
| WP_REGULATORY_CIRCUITS_OF_THE_STAT3_SIGNALING_PATHWAY | 0.48 | 1.62 | 0.01 | 0.19 |
| WP_RENIN_ANGIOTENSIN_ALDOSTERONE_SYSTEM_RAAS | 0.50 | 1.52 | 0.01 | 0.20 |
| WP_SELENIUM_MICRONUTRIENT_NETWORK | 0.43 | 1.49 | 0.01 | 0.20 |
| WP_SMALL_CELL_LUNG_CANCER | 0.40 | 1.41 | 0.01 | 0.20 |
| WP_TAMOXIFEN_METABOLISM | 0.84 | 2.15 | 0.00 | 0.18 |
| WP_TYROBP_CAUSAL_NETWORK | 0.52 | 1.70 | 0.01 | 0.18 |
| WP_VITAMIN_A_AND_CAROTENOID_METABOLISM | 0.50 | 1.51 | 0.01 | 0.20 |
